# Genetic diversity and structure of *Lolium perenne* ssp. *multiflorum* in California vineyards and orchards indicate potential for spread of herbicide resistance via gene flow

**DOI:** 10.1111/eva.12478

**Published:** 2017-04-18

**Authors:** Elizabeth Karn, Marie Jasieniuk

**Affiliations:** ^1^ University of California DavisDepartment of Plant SciencesDavisCAUSA

**Keywords:** agricultural weed, glyphosate, glyphosate resistance, herbicide, Italian ryegrass, *Lolium perenne* ssp. *multiflorum*, microsatellite markers

## Abstract

Management of agroecosystems with herbicides imposes strong selection pressures on weedy plants leading to the evolution of resistance against those herbicides. Resistance to glyphosate in populations of *Lolium perenne* L. ssp. *multiflorum* is increasingly common in California, USA, causing economic losses and the loss of effective management tools. To gain insights into the recent evolution of glyphosate resistance in *L. perenne* in perennial cropping systems of northwest California and to inform management, we investigated the frequency of glyphosate resistance and the genetic diversity and structure of 14 populations. The sampled populations contained frequencies of resistant plants ranging from 10% to 89%. Analyses of neutral genetic variation using microsatellite markers indicated very high genetic diversity within all populations regardless of resistance frequency. Genetic variation was distributed predominantly among individuals within populations rather than among populations or sampled counties, as would be expected for a wide‐ranging outcrossing weed species. Bayesian clustering analysis provided evidence of population structuring with extensive admixture between two genetic clusters or gene pools. High genetic diversity and admixture, and low differentiation between populations, strongly suggest the potential for spread of resistance through gene flow and the need for management that limits seed and pollen dispersal in *L. perenne*.

## 1 Introduction

Weedy plants pose a major problem to agricultural production causing significant crop losses worldwide and economic damages estimated to total $33 billion annually in the United States (Oerke, 2006; Pimentel, Zuniga, & Morrison, 2005). Weeds are an ongoing challenge for farmers as weed control practices exert strong selection for the evolution of weed adaptations that render the management practices less effective over time (Barrett, 1983; Owen, Michael, Renton, Steadman, & Powles, 2011; Powles & Yu, 2010). One of the best examples of this process is the evolution of resistance to herbicides. In weed populations containing phenotypic variation for susceptibility to an herbicide, those individuals with an inherited ability to survive and reproduce following an herbicide application are favored and resistance increases in the population over time (Delye, Jasieniuk, & Le Corre, 2013; Neve, Vila‐Aiub, & Roux, 2009). To date, over 470 cases of resistance in 250 species have been documented to a wide variety of herbicides worldwide (Heap, 2016).

Whether or not a weed population is able to adapt in response to management practices depends on whether that population contains the necessary genetic variation (Jasieniuk, Brule‐Babel, & Morrison, 1996; Sakai et al., 2001). Population size, standing genetic variation, selection, and gene flow with other populations all play a role in the spatial distribution of evolved adaptive traits (Delye, Jasieniuk, et al., 2013; Lawton‐Rauh, 2008). For studies of adaptation in agricultural weeds, strong selection pressures on weed populations such as tillage or herbicide application are usually known and population sizes are often large (Neve et al., 2009). Population sizes of common weeds vary across an agricultural landscape with some areas containing heavy infestations, allowing for high genetic diversity within a species across a region through the accumulation of mutations over time. In self‐pollinating weeds, populations may be genetically uniform as individuals within populations often share nearly identical highly homozygous genotypes because of repeated inbreeding, but populations are likely to differ genetically (Ward & Jasieniuk, 2009). In contrast, obligately outcrossing weeds are expected to contain high genetic diversity within populations but low genetic differentiation among populations. The amount and distribution of phenotypic and genetic variation within weed populations influence the potential for adaptation in agricultural landscapes, which are variable in both space and time as a result of habitat fragmentation due to diverse crops and associated crop and weed management practices. Ultimately, the adaptation of weed populations to a variable environment across an agricultural landscape may lead to population structuring in both selfing and outcrossing weeds.

Genetic diversity in weed populations is required for weed adaptation, but is also impacted by it as a result of strong positive selection and population bottlenecks (Neve et al., 2009). Successful herbicide applications kill 95%–99% of individuals in susceptible weed populations. This substantial reduction in population size may mean that the alleles of only a small fraction of individuals are passed on to the next generation, potentially causing some alleles to be lost by genetic drift. Alternatively, strong selection will favor selectively advantageous alleles, if present in the population, and reduce population genetic diversity. For instance, individuals which survive herbicide treatment due to heritable mechanisms will pass on their resistance‐conferring alleles to their progeny, and resistance will increase in frequency in the population over time. As the frequency of resistant individuals increases in a population, further herbicide applications will become less effective in reducing population size, leading to restoration of populations to their original size but with decreased genetic diversity. Strong selection for resistance may also be associated with a selective sweep at causative loci which not only results in the loss of susceptible alleles at the adaptive locus but also any alleles at loci in gametic disequilibrium with it (Maynard‐Smith & Haig, 1974; Menchari, Delye, & Le Corre, 2007). In summary, weed populations with a high frequency of resistant individuals are expected to contain lower genetic diversity than populations with a low frequency of resistant individuals both due to population bottlenecks while an herbicide is still effective in controlling the weed and due to selection as resistance to the herbicide evolves.

*Lolium perenne* ssp*. multiflorum* (Italian ryegrass) is an annual grass weed that causes economic losses in annual and perennial cropping systems worldwide (Preston, Wakelin, Dolman, Bostamam, & Boutsalis, 2009). *L. perenne* has an obligately outcrossing, self‐incompatible mating system with wind‐mediated pollen movement (Fearon, Hayward, & Lawrence, 1983). Populations of *L. perenne* ssp. *multiflorum* and the closely related *L. perenne* ssp. *rigidum* have repeatedly evolved resistance to several herbicides from different classes (Heap, 2016; Owen, Martinez, & Powles, 2014; Preston et al., 2009). The ability of *L. perenne* to rapidly evolve resistance to herbicides has been attributed to high genetic diversity within populations resulting from large population sizes and a self‐incompatible outcrossing mating system (Balfourier, Charmet, & Ravel, 1998; Busi & Powles, 2009). However, while the genetic diversity of cultivated and wild accessions of *L. perenne* have been reasonably well characterized (e.g., Brazauskas, Lenk, Pedersen, Stender, & Lübberstedt, 2011; Kubik, Sawkins, Meyer, & Gaut, 2001; McGrath, Hodkinson, & Barth, 2007; Wang, Dobrowolski, Cogan, Forster, & Smith, 2009), the genetic variation and structure of weedy populations in agricultural settings (crop fields, orchards, vineyards) have not been examined, to our knowledge, despite the unique demographic processes and selective pressures in agricultural systems that are likely to shape genetic diversity in weeds. To date, studies of weedy *L*. *perenne* have largely focused on characterizing herbicide resistance phenotypes and resistance levels (e.g., Jasieniuk et al., 2008; Busi & Powles, 2009, Busi, Neve, & Powles, 2013; Liu, Hulting, & Mallory‐Smith, 2016) and determining the underlying physiological and genetic mechanisms of resistance (e.g., Avila‐Garcia, Sanchez‐Olguin, Hulting, & Mallory‐Smith, 2012; Gaines et al., 2014; Ge et al., 2012; Mahmood, Mathiessen, Kristensen, & Kudsk, 2016; Yu, Abdallah, Han, Owen, & Powles, 2009). In California, a population of *L. perenne* was identified with resistance to glyphosate in 1998 (Simarmata, Kaufmann, & Penner, 2003), and glyphosate resistance was later found to have spread in perennial cropping systems of the Central Valley of California (Jasieniuk et al., 2008). In 2013, populations of *L. perenne* suspected of containing individuals resistant to glyphosate were identified in Sonoma County and Lake County in northwestern California, outside of the Central Valley, after 2 years of failed control with glyphosate.

It has been hypothesized that gene flow may spread herbicide resistance among weed populations within an agricultural landscape to a greater degree than novel mutations as rates of gene flow are generally believed to be higher than rates of mutation (Jasieniuk et al., 1996). Herbicide resistance alleles may be present in populations prior to the onset of selection pressure by an herbicide (Delye, Deulvot, et al., 2013), and may spread by gene flow even before the trait is selectively advantageous. Evidence for the spread of herbicide resistance among populations by seed dispersal has been shown in several highly self‐pollinating weed species, based on patterns of molecular marker and phenotypic variation (Okada et al., 2013, 2014; Osuna, Okada, Ahmad, Fischer, & Jasieniuk, 2011). Interestingly, however, neutral genetic and phenotypic variation in *Ipomoea purpurea*, a weed species with a mixed mating system, provided support for independent origins of resistance in multiple geographic locations (Kuester, Chang, & Baucom, 2015). In outcrossing weeds, analyses of neutral genetic variation revealed low population differentiation, and possible spread of resistance through local gene flow (Delye, Clement, Pernin, Chauvel, & Le Corre, 2010) but independent origins through novel mutations (Menchari et al., 2007).

The goal of the current study was to characterize genetic variation of northwestern California *L. perenne* populations where herbicide resistance evolution is very recent and likely ongoing. We examined the frequency of glyphosate‐resistant plants in populations across the landscape along with microsatellite marker variation to address the following questions: (i) do populations of outcrossing weeds contain high genetic diversity and is this diversity reduced in populations with a high frequency of glyphosate‐resistant individuals, (ii) is there evidence of genetic structuring and differentiation among populations of this widespread weed across an agricultural landscape, and (iii) is there potential for spread of resistance alleles across the landscape through gene flow?

## 2 Materials and methods

### 2.1 Population sampling

To determine whether glyphosate‐resistant individuals are present in *L. perenne* ssp. *multiflorum* populations in northwest California, we sampled 13 orchards and vineyards in 2013 from Sonoma County and Lake County (Table 1) in the general regions where growers had reported difficulty controlling plants with glyphosate to farm advisors and in surrounding areas where populations may be experiencing gene flow with resistant plants. One population identified as resistant to glyphosate from Butte County was also sampled to serve as a comparison with an area which had evolved resistance greater than 10 years ago (Jasieniuk et al., 2008; Simarmata et al., 2003). Within each population, young leaf tissue and panicles with mature seed were collected from each of 30–40 individuals at least one meter apart from one another while walking randomly selected tree or vine rows. Leaf tissue was transported to the laboratory for DNA extraction. Seed panicles were stored in paper envelopes for 3 months to allow seeds to after‐ripen and overcome dormancy before planting and testing plants for resistance to glyphosate.

**Table 1:** *Lolium perenne* ssp. *multiflorum* populations sampled for this study

Pop ID	Cropping system	County	Latitude (N)	Longitude (W)	N_S_	N_G_	N_P_	%R
1	Orchard	Butte	39.80	−121.98	32	23	128	73.8
2	Vineyard	Sonoma	38.23	−122.52	30	28	128	9.7
3	Vineyard	Sonoma	38.24	−122.42	37	29	171	22.5
4	Vineyard	Sonoma	38.24	−122.36	33	18	212	29.1
6	Vineyard	Sonoma	38.359	−122.502	34	31	123	22.7
7	Vineyard	Sonoma	38.214	−122.457	33	32	65	32.1
8	Vineyard	Sonoma	38.587	−122.829	31	31	176	26.6
9	Vineyard	Sonoma	38.662	−122.825	33	32	150	31.6
10	Vineyard	Sonoma	38.673	−122.811	32	31	55	35.2
11	Vineyard	Sonoma	38.761	−122.976	41	41	166	40.6
12	Vineyard	Lake	38.989	−122.821	20	19	91	85.1
13	Orchard	Lake	38.997	−122.834	36	36	186	89.0
14	Orchard	Lake	38.996	−122.84	31	31	153	87.9
15	Orchard	Lake	39.086	−122.943	30	30	145	20.6

N_S_, number of individuals sampled for leaf tissue and seeds from each population; N_G_, number of individuals genotyped; N_P_, number of progeny phenotyped for response to glyphosate; % R, percentage of individuals surviving treatment with glyphosate at 1681 g a.e. ha^−1^.

### 2.2 Phenotyping plant response to glyphosate

Eight seeds from each sampled plant were germinated on moistened filter paper in petri dishes at 20°C and a 12‐hr photoperiod. Germinated seedlings were transplanted into 8 × 8 cm square pots filled with UC soil mix (sand, compost, and peat in 1:1:1 ratio with 1.8 kg/m^3^ dolomite) with two seedlings per pot and grown in the glasshouse at 27/15°C with ambient light conditions. At the tillering stage, individual plants were divided into genetically identical clones following the method described by Boutsalis (2001) and grown in the glasshouse to the 2–3 leaf stage. One clone of a genotype was treated with water, which served as a control. The second clone was treated with glyphosate (Roundup PowerMax, Monsanto, St. Louis, MO) at the rate of acid equivalent 1,681 g/ha, which is twice the recommended (label) field rate for the control of annual *Lolium perenne* plants under six inches tall. All treatments were applied in an enclosed cabinet track sprayer equipped with an 8002E nozzle (TeeJet, Spraying Systems Co., Wheaton, IL) delivering 200 L/ha. Three weeks after glyphosate treatment, we scored each plant as alive or dead and characterized the percentage of resistant plants in each population by the percentage of plants surviving glyphosate treatment of the total number of plants treated. Plants from a previously characterized susceptible reference seed collection (Jasieniuk et al., 2008) were included during each herbicide application to confirm herbicide activity.

### 2.3 Genotyping plants using microsatellite markers

DNA was extracted from collected leaf tissue following the CTAB method (Doyle & Doyle, 1987), then quantified, and diluted to 25 ng/μl. We genotyped individuals at 12 polymorphic loci using 11 microsatellite primer pairs (Table 2), which included b1b1, b1b3, b3b1, b3b8, b3c5, b1a8, b4d3, b5d12 (Lauvergeat, Barre, Bonnet, & Ghesquiere, 2005), pr3 (Kubik et al., 2001), and 14C9, 44A7 (King et al., 2008). Primer pair 14C9 amplified two loci, called 14C9‐1 and 14C9‐2. Two sets of alleles, with one or two alleles in each of the two regions amplified by this primer pair, were observed in all individuals genotyped at these loci. For all primer pairs, forward primers labeled with either 6‐Fam or Hex (Integrated DNA Technologies, Coralville, IA) were used in PCRs consisting of 25 ng DNA template, 1 ×  Qiagen PCR buffer (Valencia, CA), 0.25 mM additional MgCl_2_, 0.4 μM forward and reverse primers, 0.125 mM DNTPs, and 0.5 units Taq polymerase. The PCR program consisted of an initial denaturing period of 3 min at 94°C, followed by 30 cycles of 1 min at 94°C, 1 min at x°C, 2 min at 72°C, and a final extension of 10 min at 72°C, where x is a primer‐dependent annealing temperature (Table 2). PCR products were multiplexed into six pairs of PCR product and separated using an ABI 3100 Genetic Analyzer (Applied Biosystems, Foster City, CA) with GENESCAN 400HD as an internal size standard (Applied Biosystems). Fragments were sized with GeneMapper 3.7 (Applied Biosystems). Genotypes were also inspected manually.

**Table 2:** Characteristics and sources of 12 microsatellite loci used to genotype *Lolium perenne* ssp. *multiflorum*. Microsatellite markers were selected based on polymorphism and consistent amplification of alleles

Marker		Forward and Reverse Primers	Ta	Repeat Motif	Source
b1b1	f	CAGGTCCAGCGCTAGTGTTA	57	(CT)4(CA)2N	Lauvergeat et al. (2005)
	r	GAGGTGTGGTGCTGGGATAG		136(AC)7	
b1b3	f	AGGTGTCCTGTTGCTTTGGA	57	(TG)7	Lauvergeat et al. (2005)
	r	TTTACCCCCAGGGATCAAAT			
b3b1	f	TTTCCCTGGGATAGCGTTAG	57	(TG)10	Lauvergeat et al. (2005)
	r	TTAGCATAAACAGATGAAGCATAAC			
b3b8	f	TGTCATGTCCGCTGTCTACG	57	(CA)10	Lauvergeat et al. (2005)
	r	GAGAGTGGGCGATCATCTTC			
b3c5	f	TGTCATGTTCAGAAAGTGCG	55	(GT)8	Lauvergeat et al. (2005)
	r	TGTCCACATAAATGCACCTCA			
b1a8	f	GACTTTCAGGCATCGGTCAT	57	(TG)7	Lauvergeat et al. (2005)
	r	CCCAGCTCCATTCTTAATGC			
b4d3	f	ATTGATGGTGCCACTCCTCT	53	(CA)7	Lauvergeat et al. (2005)
	r	ATGGACAAAGCAGGGGTTC			
b5d12	f	GAATCCTCGATGTGGGCTAC	53	(GT)5	Lauvergeat et al. (2005)
	r	TAAAACGGAACCACCCATTC			
pr3	f	GTATAGTACCCATTCCGT	53	(CA)22	Kubik et al. (2001)
	r	GCCGCCCTGCCATGCTG			
14C9‐1	f	AATGATGGCACGGAGCAATCG	50	(CT)22	King et al. (2008)
	r	CTGTAATTCCAGGTCACTACC			
14C9‐2	f	AATGATGGCACGGAGCAATCG	50	CT	King et al. (2008)
	r	CTGTAATTCCAGGTCACTACC			
44A7	f	CACGTAGAAGCCACACTTTAC	50	(CT)60	King et al. (2008)
	r	GTCACATTCCATTCACTTCCG			

Ta, annealing temperature.

Primer pair 14C9 amplified two independent microsatellite loci (see Results section).

### 2.4 Microsatellite marker error rate

To assess genotyping errors, we tested for null alleles or genotyping stutter using Microchecker version 2.2.3 (Van Oosterhout, Hutchinson, Wills, & Shipley, 2004). Because null alleles were detected for several loci, FreeNa software (Chapuis & Estoup, 2007) was used to estimate the frequency of null alleles and their effect on F_ST_ estimates using the standard F_ST_ estimation method and the unbiased adjusted “excluding null alleles” F_ST_ (ENA) method with 1,000 bootstrap replicates. We tested standard and adjusted F_ST_ values for differences using Student's t‐test.

### 2.5 Genetic diversity and structure

To estimate the allelic diversity of each locus, we calculated the total number of alleles detected at each locus (N_A_) using F_STAT_ software version 2.9.3.2 (Goudet, 1995). Wright's inbreeding coefficient (F_IS_) was estimated for each of the 14 populations and averaged over populations for each locus, and Wright's fixation index (F_ST_) was calculated over the 14 populations by locus, also in F_STAT_. Statistical significances of F_IS_ and F_ST_ were determined with 1,000 permutations. Observed heterozygosity (H_O_) and total gene diversity or expected heterozygosity (H_E_) at each locus were calculated using Genepop on the Web software version 4.2 (option 5, suboption 1; Raymond & Rousset, 1995; Rousset, 2008).

To estimate genetic diversity within each population across all 12 loci, we calculated the average number of alleles detected per locus (N_A_), mean allelic richness (A_R_) defined as the number of alleles rarified to the smallest population sample size*,* and Wright's inbreeding coefficient (F_IS_) in F_STAT_. Statistical significance of F_IS_ was determined with 1,000 permutations. We also calculated H_O_ and H_E_ for each population in Genepop on the Web (option 5, suboption 1). We did not estimate population differentiation based on the statistic R_ST_ because stepwise mutations at microsatellite loci likely contribute relatively little to genetic differentiation between recently founded populations, such as those of weedy *Lolium perenne* (Kalinowski, 2002).

Departures from Hardy–Weinberg proportions per locus and per population were tested using the Hardy–Weinberg exact test with default Markov chain parameters in Genepop on the Web (option 1, suboption 3). To test for linkage disequilibrium between each pair of loci in each population, we performed a pairwise log likelihood ratio test for disequilibrium in Genepop on the Web (option 2, suboption 1) with default Markov chain parameters. To detect recent changes in effective population sizes or population bottlenecks, we performed a one‐tailed Wilcoxon sign‐rank test for heterozygote excess compared to that expected under a drift–mutation model using Bottleneck software version 1.2.02 (Cornuet & Luikart, 1997; Piry, Luikart, & Cornuet, 1999). A sequential Bonferroni correction was applied to adjust significance levels for multiple comparisons (Rice, 1989).

To investigate the spatial structuring of genetic variation among populations and counties, we performed multiple distance‐based analyses using GenAlEx 6.5 (Peakall & Smouse, 2006, 2012). First, we calculated a matrix of genetic distances, based on Nei's D (Nei, 1978), between each pair of populations with GenAlEx software interpolating for missing data, and then used the pairwise genetic distances in the following analyses. We performed a principal coordinate analysis (PCA) using the standardized covariance method in GenAlEx to assess whether populations located within the same county and/or geographically near each other were also genetically similar and thus may share an evolutionary history. To test for isolation by distance (IBD) and determine whether genetic structuring correlates with geographic structuring between populations, we conducted a Mantel test (Mantel, 1967) in GenAlEx with 1,000 permutations between the matrix of pairwise genetic distances described above and a matrix of pairwise geographic distances calculated using the GPS coordinates (latitude and longitude) of each population. We also performed a hierarchical analysis of molecular variance (AMOVA; Excoffier, Smouse, & Quattro, 1992) in GenAlEx, which examined the distribution of genetic variation at five hierarchical levels: among counties within the total sample, among populations within those counties, among populations in the total sample, among individuals within populations, and among individuals within the total sample. The AMOVA was performed with 1,000 permutations.

To assess population structure and determine the degree of admixture among populations, we used a model‐based Bayesian clustering algorithm in STRUCTURE software version 2.3.4 (Pritchard, Stephens, & Donnelly, 2000). STRUCTURE infers genetic clusters or populations based on the multilocus genotypes of all individuals, independent of sampled location, by probabilistically assigning individuals to a cluster or jointly to multiple clusters if their genotypes indicate admixture between clusters, while simultaneously maximizing Hardy–Weinberg equilibrium and minimizing linkage disequilibrium within those clusters. STRUCTURE analysis was performed with a burn‐in period of 1,000,000 iterations followed by 1,000,000 iterations for the number of genetic clusters (*K*) ranging from *K* = 1 to *K* = 12. Five independent runs at each value of *K* were performed using the population admixture model for potentially interbreeding populations and correlated allele frequencies. Likelihood values of ln P(D) were assessed for each run. The most likely value of *K* was inferred using the ΔK method (Evanno, Regnaut, & Goudet, 2005) in STRUCTURE Harvester online (Earl & von Holdt, 2012). Each individual's probability of assignment to each cluster (*q*), also interpreted as the proportion of an individual's genome that originated in each cluster (Pritchard et al., 2000), was visualized for all individuals using Distruct software version 1.1 (Rosenberg, 2004). To examine substructuring within genetic clusters, the multilocus genotypes of individuals with *q* > 0.6 to a cluster were analyzed independently, as suggested by Evanno et al. (2005), using the same parameters as above.

## 3 Results

### 3.1 Plant response to glyphosate

**Figure 1:**
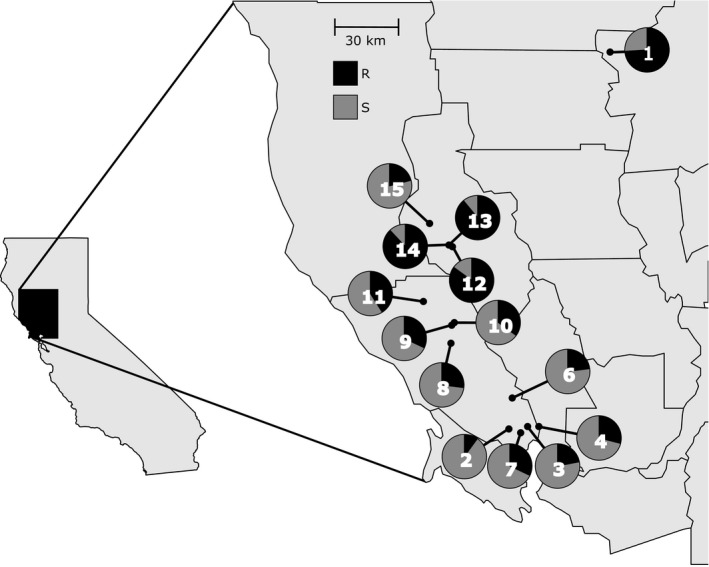
Geographic distribution of the populations sampled for this study in northwest California. Circles indicate the proportion of glyphosate‐resistant (black) and glyphosate‐susceptible (gray) individuals in each population, based on glasshouse screening of plants grown from field‐collected seeds. Numbers are the population IDs (see Table 1)

Within sampled populations of *L. perenne* ssp. *multiflorum*, resistance to glyphosate, estimated as the percentage of individuals surviving glyphosate treatment per population, varied from 9.7% to 89.0% (Figure 1, Table 1). The Butte County population sampled from the area where glyphosate resistance was first reported in California (Simarmata et al., 2003) contained 73.8% resistant individuals. In Lake County, three populations (populations 12, 13, and 14) from an area where growers reported possible resistance contained 85%–89% resistant individuals, while a population (population 15) bordering the area contained 21% resistant individuals. In Sonoma County, populations show a gradient of survivorship ranging from 9.7% survivorship in the southern end to 40.6% in the northern end of the county (Table 1).

### 3.2 Genetic diversity and structure

#### 3.2.1 Genetic diversity of microsatellite loci

Information on null alleles for each locus can be found in the Data S1 section. We detected 259 distinct alleles in 412 individuals of *L. perenne* ssp. *multiflorum* across 12 microsatellite loci. The total number of alleles detected per locus ranged from 8 to 56 (Table 3), and all loci were polymorphic in each population. Observed heterozygosity (H_O_) ranged from 0.147 to 0.754, while expected heterozygosity (H_E_) varied from 0.678 to 0.899. All loci revealed a reduction in observed heterozygosity compared to expected heterozygosity. Correspondingly, values of the inbreeding coefficient, F_IS_, were statistically significant for all loci and ranged from 0.068 to 0.824. Because a reduction in heterozygosity compared to that expected under Hardy–Weinberg conditions was observed across all loci regardless of whether null alleles were detected, it is unlikely that null alleles are the major cause of heterozygosity deficits. Per locus estimates of F_ST_ ranged from 0.006 to 0.088, indicating little genetic differentiation among populations at each locus (Table 3). Among 923 pairwise comparisons of loci and populations, linkage disequilibrium was detected in only eight locus‐by‐population test combinations following Bonferroni correction (Table S2). Linkage disequilibrium was never detected between the same pair of loci twice, consistent with independently segregating loci. One microsatellite primer pair, 14C9, was found to amplify two separate loci (14C9‐1 and 14C9‐2) with nonoverlapping allele sizes (Table 2, Table 3). Both loci were scored and treated as separate microsatellite loci in data analyses. At locus 44A7, 56 alleles were identified ranging in size from 131 to 278 base pairs (Table 3). Despite the large range of allele sizes, no division of alleles into separate size classes that might indicate amplification of multiple loci was detected, and all individuals contained either one or two alleles as expected for diploids genotyped at a single microsatellite locus.

**Table 3:** Genetic diversity detected at 12 microsatellite loci in 412 individuals of *Lolium perenne* spp. *multiflorum*

Marker	N_A_	Allele sizes (bp)	% missing	H_E_	H_O_	F_IS_	F_ST_
b1b1	17	290–328	5.3%	0.861	0.382	0.541**	0.049*
b1b3	18	207–246	2.7%	0.705	0.392	0.435**	0.034*
b3b1	25	253–321	10.4%	0.878	0.461	0.476**	0.015
b3b8	26	292–335	2.9%	0.899	0.578	0.339**	0.020*
b3c5	16	113–146	4.6%	0.794	0.441	0.395**	0.088*
b1a8	26	237–303	1.2%	0.874	0.754	0.142**	0.015
b4d3	13	286–320	13.6%	0.788	0.388	0.492**	0.042
b5d12	8	102–116	0.7%	0.678	0.598	0.068*	0.051*
pr3	19	122–170	12.6%	0.786	0.352	0.538**	0.013
14C9‐1	22	208–256	0.5%	0.867	0.640	0.263**	0.006
14C9‐2	13	353–393	12.9%	0.825	0.147	0.824**	0.011
44A7	56	131–278	0.0%	0.826	0.385	0.593**	0.015*

N_A_, total number of alleles detected; allele sizes, range of PCR product sizes (bp); % missing, % missing data at each locus; H_E_, expected heterozygosity; H_O_, observed heterozygosity; F_IS_, Wright's inbreeding coefficient; F_ST_, Wright's fixation index were averaged over 14 populations sampled in California. *p* values: *p < .01, **p < .001.

#### 3.2.2 Genetic diversity of populations

Within populations, the average number of alleles detected per locus (N_A_) was high ranging from 8.7 to 12.1. Correspondingly, allelic richness (A_R_) ranged from 7.7 to 9.2 with an average of 8.4 over all populations (Table 4). Expected heterozygosity (H_E_) ranged from 0.74 to 0.81 among populations, whereas observed heterozygosity (H_O_) ranged from 0.40 to 0.52, indicating a heterozygote deficiency relative to Hardy–Weinberg expectations in all populations. Accordingly, values of the inbreeding coefficient F_IS_ for populations were high ranging from 0.374 to 0.475 (Table 4). Population bottlenecks as indicated by heterozygote excess relative to expectations under a drift–mutation model were detected in seven populations (Table 4).

**Table 4:** Genetic diversity within populations of *L. perenne* ssp. *multiflorum* based on variation at 12 microsatellite loci

Pop ID	N_G_	N_A_	A_R_	H_E_	H_O_	F_IS_	B
1	23	8.75	7.68	0.771	0.454	0.431**	0.0003*
2	28	10.92	8.72	0.776	0.482	0.396**	0.0052
3	29	11.08	8.81	0.788	0.431	0.467**	0.0212
4	18	9.42	8.72	0.805	0.517	0.384**	0.0052
6	31	11.75	8.87	0.800	0.441	0.462**	0.0017*
7	32	11.25	8.62	0.792	0.427	0.475**	0.0052
8	31	10.75	8.38	0.782	0.501	0.374**	0.0023*
9	32	11.25	8.54	0.786	0.490	0.391**	0.0008*
10	31	11.00	8.82	0.774	0.443	0.442**	0.0017*
11	41	10.92	7.58	0.738	0.396	0.474**	0.0052
12	19	8.67	8.18	0.797	0.498	0.400**	0.0012*
13	36	12.08	9.22	0.809	0.459	0.445**	0.0261
14	31	9.58	7.71	0.757	0.470	0.395**	0.0006*
15	30	9.58	7.82	0.794	0.430	0.473**	0.0319

N_G_, number of individuals genotyped; N_A_, average number of alleles detected per locus; A_R_, mean allelic richness; H_E_, expected heterozygosity; H_O_, observed heterozygosity; F_IS_, Wright's inbreeding coefficient, and B, the *p* value of Wilcoxon sign‐rank test for genetic bottleneck. *p* values: *p < .05 following Bonferroni correction. **p < .001.

Three populations (1, 12, and 14) containing a high frequency of resistant individuals (% R > 70%) all show a lower than average allelic richness (A_R_ = 7.7, 8.1, and 7.7, respectively) (Table 4), which might indicate that populations with a high frequency of resistant individuals have lower genetic diversity. Bottlenecks were detected in these three populations (Table 4). However, the population (population 13) containing the highest frequency of glyphosate‐resistant individuals (89% R) (Table 1) also had the highest number of alleles detected per locus (N_A_ = 12.1) and the highest allelic richness (A_R_ = 9.2), and no bottleneck was detected. Correspondingly, there is no significant correlation between frequency of resistant plants within populations and allelic richness (Spearman's rank coefficient ρ = −0.22, *p* = .449). If population 13 is removed from the analysis, the correlation is stronger but still not significant (Spearman's rank coefficient ρ = −0.525, *p* = .065).

### 3.3 Population structure

**Figure 2:**
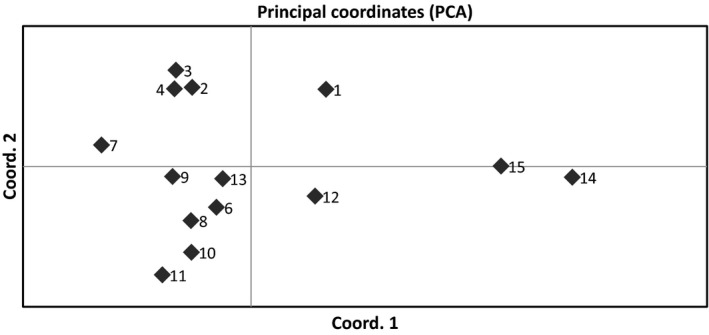
Principal coordinate analysis (PCoA) of pairwise genetic distances between populations. The first two axes explain 32.2% and 19.1% of genetic variation

A principal coordinate analysis (PCA) of genetic distances between populations revealed differentiation among populations with grouping of some populations by geographic origin (Figure 2). Most populations from the southern end of Sonoma County (2, 3, and 4) group tightly together, while populations from the central and northern parts of Sonoma County (6, 8, 9, 10, and 11) along with population 13 from Lake County group together. Populations located in Lake County (12, 13, 14, and 15) are more genetically distant from each other and do not group tightly together. The percentage of variation explained by the first two axes are 32.2% and 19.1%. A Mantel test revealed a weak, nonsignificant correlation between genetic and geographic distances (slope = 0.0105, R^2^ = 0.14, *p* = .468), indicating that the genetic differentiation observed among populations is not related solely to geographic isolation.

As population clustering by county was not explained by geographic distance, an AMOVA was conducted to determine how much of the genetic variance could be attributed to county or population differences. The AMOVA revealed low, but significant, genetic differentiation among counties (F_RT_ = 0.018, *p* = .001), among populations within counties (F_SR_ = 0.018, *p* = .001), and among populations within the total sample (F_ST_ = 0.036, *p* = .001) (Table 5). Genetic differences among individuals within populations (F_IS_ = 0.466, *p* = .001) and among individuals within the total sample (F_IT_ = 0.485, *p* = .001) are high. Because of large genetic differences among individuals, 45.6% of the genetic variation is distributed among individuals within populations and 47.4% among individuals in the total sample, with 1.8% of genetic variation distributed between counties, 1.8% among populations within counties, and 3.5% among populations in the total sample, based on the F‐statistic for the corresponding measure.

**Table 5:** Analysis of molecular variance (AMOVA) results showing F‐statistics for codominant allelic data for genetic variation distributed among five hierarchical levels

Effect	F‐statistic	*F*	*p*	% of total genetic variation
Among counties in total sample	F_RT_	0.018	.001	1.8
Among populations in counties	F_SR_	0.018	.001	1.8
Among populations in total sample	F_ST_	0.036	.001	3.5
Among individuals in populations	F_IS_	0.466	.001	45.6
Among individuals in total sample	F_IT_	0.485	.001	47.4

**Figure 3:**
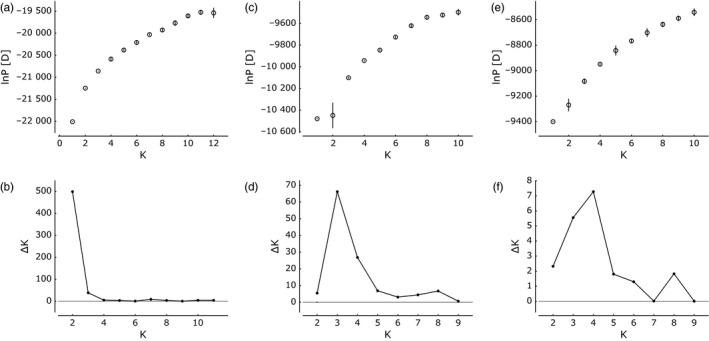
Bayesian clustering analysis (STRUCTURE, Pritchard et al., 2000) of *Lolium perenne* plots of (a, c, e) the log likelihood ln P[D] for five runs at each value of *K*, and (b, d, f) the second order of change in ln P[D], ΔK, as a function of the number of clusters or gene pools, *K*, from the analysis of all samples (a, b) and subclustering analysis of individuals assigning with *q* > 0.6 to cluster 1 (c, d) and to cluster 2 (e, f) within the global analysis

**Figure 4:**
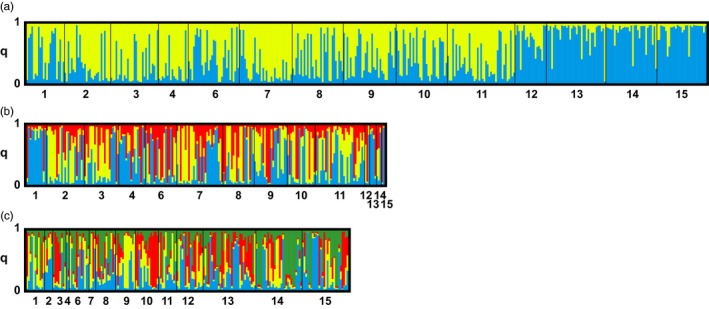
Assignment of 412 individuals of *L. perenne* ssp. *multiflorum* to the genetic clusters inferred by Bayesian clustering analysis (STRUCTURE). Each vertical bar corresponds to a distinct individual and its probability of assignment, *q*, to each cluster. (a) *K* = 2, the most likely number of genetic clusters for the global data set, (b) *K* = 3, the most likely number of subclusters among individuals assigning with *q* > 0.6 to cluster 1, and (c) *K* = 4, the most likely number of subclusters among individuals assigning with *q* > 0.6 to cluster 2

STRUCTURE analysis (Pritchard et al., 2000) was used to further examine genetic structuring. STRUCTURE revealed increasing values of ln P(D) with increasing *K* values ranging from 1 to 12 with no clear maximum likelihood (Figure 3a). ΔK (Evanno et al., 2005) clearly showed the highest value at *K* = 2, indicating two genetic clusters (Figure 3b). The proportion of the genome, as represented by the 12 microsatellite loci, that assigns to each cluster, *q*, was calculated for each individual. Individuals assigning to cluster 1 with *q* > 0.7 were comprised of some individuals from Sonoma County and Butte County and a few individuals from Lake County, while most individuals from Lake County and some from Sonoma County and Butte County assigned to cluster 2 (Figure 4a). While individuals from Sonoma County and Butte County assigned to both genetic clusters, individuals from Lake County assigned highly to cluster 2. All populations contained some individuals that assigned partially to each cluster (*q* < 0.6) indicating admixture between genetic clusters (Figure 4a).

There is no apparent pattern of genetic structuring based on whether the individuals originated from field populations that were predominantly resistant or susceptible to glyphosate (Table 1). The majority of individuals from populations 12, 13, and 14, where the frequency of glyphosate resistance was 85%–89%, assigned to cluster 2 with *q* > 0.7, but most individuals from population 15 where resistance frequency was 21% also assigned to the same cluster. Of the individuals from population 1 genotyped, 48% and 52% assigned to clusters 1 and 2, respectively, but 77% of individuals phenotyped were resistant to glyphosate, indicating that glyphosate‐resistant individuals likely assign to both clusters.

To examine patterns of hierarchical population structure, individuals assigning to each cluster with *q* > 0.6 were separated and analyzed independently. STRUCTURE analysis of each cluster revealed evidence of subclustering, with *K* = 3 within cluster 1 and *K* = 4 within cluster 2 (Figure 3c–f). Among individuals that assigned to cluster 1, most individuals assigned highly to one of the three subclusters (Figure 4b). However, there is little apparent geographic substructuring of genetic variation, with the exception that most individuals from Butte County assigned to subcluster 1. Among individuals that assigned to cluster 2, most individuals were admixed, assigning to multiple subclusters (Figure 4c).

## 4 Discussion

### 4.1 High genetic variation observed in weedy *L. perenne* regardless of resistance frequency

Our analyses indicate very high genetic diversity within populations of *L. perenne* ssp. *multiflorum* as expected based on the biology of this widespread obligately outcrossing species. However, given the large number of detected alleles and the allele frequencies observed, a higher level of heterozygosity was expected than was observed across all populations (Table 4). While some of this reduction in heterozygosity may be due to null alleles that do not amplify during PCR creating the false appearance of homozygous individuals, this would result in reduced observed heterozygosity only in loci with a high frequency of null alleles. However, a reduction in observed heterozygosity is present across all loci, leading to the conclusion that there is a biological cause for the lower than expected heterozygosity (Table 3). This could be due to a very recent or ongoing population bottleneck in *L. perenne* populations due to control by glyphosate or other weed management practices. Seven of the 14 sampled populations show evidence of a past bottleneck. These seven populations may be located in fields with more intensive weed management and thus have undergone a stronger bottleneck. In populations with no detected bottleneck, extensive gene flow with other populations could have restored genetic diversity and erased the genetic signature of a past population bottleneck. Bottlenecks are only detectable if they are either very strong or very recent (Luikart & Cornuet, 1998), although the bottleneck imposed by intense selection by glyphosate and the evolution of glyphosate resistance likely has been recent, probably occurring within the last 20 years, that is 20 generations, based on when resistance to glyphosate first was identified in *Lolium* in the United States and worldwide (Powles, Lorraine‐Colwill, Dellow, & Preston, 1998; Simarmata et al., 2003). Although resistance alleles may have been present at low frequencies previous to 20 years ago, they did not rise to frequencies high enough to be problematic until glyphosate use in agriculture intensified, following the introduction of inexpensive generic formulations of the herbicide and widespread adoption of reduced‐ or no‐tillage cropping systems and glyphosate‐resistant transgenic crops.

Populations which have undergone recent population bottlenecks and strong selective sweeps for adaptive traits may be expected to contain lower genetic diversity than populations, which have not yet adapted to the selection pressure. Populations with a high frequency of resistant individuals would be expected to contain lower genetic diversity than those with a lower frequency of resistant individuals. However, *L. perenne* populations with a high frequency of resistant plants (>70%) still contained high levels of observed genetic diversity, despite evidence of a population bottleneck in some populations. The lack of correlation between the frequency of resistant plants within populations and the fixation index (F_IS_) (Spearman's rank coefficient ρ = −0.02, *p* = .93), within‐population genetic diversity (H_E_) (Spearman's rank coefficient ρ = −0.13, *p* = .67), and allelic richness (A_R_) (Spearman's rank coefficient ρ = −0.22, *p* = .449) suggests that weed population size and genetic diversity may have been influenced by other weed control practices or environmental conditions, in addition to treatment with glyphosate. Herbicide rotations and mixtures, tillage, mowing, and pest and environmental pressures present variable selection pressures, which may reduce genetic diversity in all populations, making it difficult to detect reductions in diversity in populations with a high frequency of glyphosate‐resistant individuals. Repeated introductions of *L. perenne* seeds from other fields may serve to increase within‐population genetic diversity and compensate for alleles lost through selective sweeps and bottlenecks. It is also possible that less‐intensively managed *L. perenne* plants growing in roadsides or noncrop areas near the sampled populations may be experiencing less intense selection and subsequently a less intense population bottleneck and that gene flow through pollen exchange with these plants may restore some genetic diversity in populations undergoing selection for resistance.

The genetic diversity observed in weedy populations of *L. perenne* was higher than that observed in other herbicide‐resistant agricultural weeds. Populations of another outcrossing grass weed species, *Alopecurus myosuroides*, in France with evolved resistance to ACCase‐inhibiting herbicides had lower values (0.246 and 0.240) of expected heterozygosity based on AFLP markers (Delye et al., 2010; Menchari et al., 2007; respectively), and populations of a glyphosate‐resistant weed with a mixed mating system, *Ipomoea purpurea*, also had lower total genetic diversity (H_E_ = 0.304) based on microsatellite markers (Kuester et al., 2015). Populations of species with an outcrossing mating system would be expected to have higher genetic diversity than those with predominantly self‐pollinating mating systems. Glyphosate‐resistant populations of the two closely related selfing species *Conyza canadensis* and *Conyza bonariensis* in California displayed a wide range of genetic diversities (H_E_ = 0.0–0.45 and H_E_ = 0.009–0.513, respectively) based on microsatellite markers (Okada et al., 2013, 2014).

While genetic diversity observed in weedy *L. perenne* populations was higher than that observed in other species of weeds, the amount of genetic diversity detected in this study was similar to that observed in most other studies of *L. perenne*. Average observed heterozygosity across loci in weedy populations (H_O_ = 0.46) was similar to values observed (H_O_ = 0.40 and H_O_ = 0.44) in some studies of perennial cultivars (Brazauskas et al., 2011 and Wang et al., 2009; respectively) but lower than another study of perennial *L. perenne* cultivars (H_O_ = 0.62) (Kubik et al., 2001) based on microsatellite markers. Expected heterozygosity in wild European *L. perenne* populations ranged from 0.233 to 0.359 based on AFLP markers (McGrath et al., 2007). The high number of alleles detected per locus averaged across populations (N_A_ = 10.5) in California populations was also similar or higher than that seen in studies of *L. perenne* cultivars using some of the same microsatellite markers (N_A_ = 19.4, 13.3, and 9.9; Kubik et al., 2001; Wang et al., 2009; Brazauskas et al., 2011, respectively). Interestingly, the forage cultivars of *L. perenne* do not show lower genetic diversity than their wild and weedy relatives despite many generations of breeding. Rather, *L. perenne* seems to display a very high level of genetic diversity, regardless of origin.

### 4.2 Spatial and genetic structuring of populations

Glyphosate‐resistant plants were detected in *L. perenne* populations from all sampled areas (Table 2). The Butte County population, located near where glyphosate resistance was first detected in California, contained a high proportion of resistant individuals (Figure 1). In Lake County, the high frequency of resistant plants in populations 12–14 was consistent with grower reports of increased difficulty controlling plants, with a lower frequency further away. In Sonoma County, a very low frequency of resistant individuals was observed in the southern end of the county, with moderate frequency in the northern end. The frequency of individuals surviving glyphosate treatment in Sonoma County was substantially lower than the other areas despite similar reports of weed control failure from growers. Glyphosate has been used for decades as the primary herbicide for weed control in orchards and vineyards, and noncrop areas of California. The appearance of glyphosate resistance is an adaptive response to the widespread and repeated use of glyphosate as a weed management strategy.

The observed microsatellite variation indicates genetic structuring of *L. perenne* populations in northwest California. Principal coordinate analysis of genetic distances indicates that populations located close to each other tend to be genetically similar, as is seen in the grouping of most populations in southern Sonoma County and of populations in northern Sonoma County together with population 13 (Figure 2). However, geographic proximity does not necessarily indicate genetic similarity. The principal coordinate analysis (Figure 2) and pairwise F_ST_ (Table S3) both reveal larger genetic distance between populations within Lake County than between populations within Sonoma County. This indicates that despite the relatively larger geographic distances, Sonoma County populations are closely related to each other due to either higher genetic exchange between them or less differentiation over time, possibly related to the relative homogeneity of the vineyard cropping system across Sonoma County compared to the mix of perennial crops grown in Lake County. Differences in water availability in primarily drip‐irrigated vineyards compared to sprinkler‐irrigated orchards may lead to local adaptation or phenological differences due to water stress may contribute to population differentiation in Lake County populations. The lack of correlation between genetic distance and geographic distance found by a Mantel test indicates that genetic distance between populations is not due mainly to factors associated with isolation by distance, but to some other factor such as local adaptation or differences in the strength of selection pressures across the landscape. Extensive long‐distance gene flow may also erode the relationship between genetic distance and geographic distance, especially if gene flow is not homogeneous across the entire range. An analysis of molecular variance indicates a significant amount of genetic variation is distributed at the county and population level (Table 5). However, both county and population differences are outweighed by the high genetic variation among individuals, as might be expected in this highly diverse outcrossing species (Brazauskas et al., 2011).

Bayesian clustering STRUCTURE analysis indicates the presence of two distinct gene pools or genetic clusters (Figure 3a). While the individuals assigning to cluster 1 are primarily from Butte County and Sonoma County, individuals assigning to cluster 2 come from all three counties and include almost all individuals from Lake County (Figure 4a). This indicates that there is little admixture between individuals in Lake County and individuals in the other sampled areas or that *L. perenne* has been introduced into the region too recently for substantial admixture to have occurred. In the subclustering analysis, there is little apparent spatial structure among individuals assigning to subclusters (Figure 4b,c). Many individuals had admixed genomes assigning partially to multiple subclusters, indicating little differentiation or high gene flow between individuals assigning to these subclusters.

### 4.3 Evolution of resistance and potential for spread of resistance alleles

Together, data on spatial patterns of population structuring and frequencies of glyphosate‐resistant phenotypes allow comparison of hypotheses regarding single or multiple evolutionary origins and subsequent spread of glyphosate resistance in *L. perenne* populations in northwest California. Populations with moderate frequencies of resistant individuals in Sonoma County (30% > R > 80%, populations 7, 9, 10, 11) contain mostly individuals that assign to cluster 1 with *q* > 0.7, while populations with high frequencies of resistant individuals (R > 80%, populations 12, 13, 14) contain a large proportion of individuals that assign to cluster 2 with *q* > 0.7 (Figure 4a, Table 2). This suggests that glyphosate resistance has likely evolved independently in individuals that assign to each cluster. Unfortunately, it was not possible to genotype and phenotype the same individuals, which would have allowed stronger inference. In addition, the high genetic diversity observed among individuals within populations and the low percentage of genetic variation observed among populations make it difficult to infer single or multiple origins of resistance using only neutral genetic variation. However, our sequencing of the gene encoding glyphosate's target enzyme in resistant and susceptible plants confirms multiple target‐site mutations, which have previously been identified to cause resistance to glyphosate, and thus multiple independent origins of glyphosate resistance (Karn & Jasieniuk, In review), in agreement with the results of STRUCTURE analysis in this study. There is also evidence that some plants are resistant due to a mechanism other than target‐site mutations, indicating that multiple mechanisms of resistance are present in the region resulting from at least one additional independent origin of resistance (Karn and Jasieniuk, in review). It is possible that in the future, additional novel mutations may result in yet more independent origins of resistance.

STRUCTURE analysis revealed potential for future spread of resistance alleles through gene flow. Many admixed individuals with genotypes that assigned partially to each cluster were identified (Figure 4). While the majority of individuals assign highly to a single cluster, the considerable number of admixed individuals in many populations indicates that gene flow is common. Localized gene flow between populations located near each other may be pollen‐mediated, and pollen movement over distances of 3 km has been documented in *L. perenne* ssp. *rigidum* (Busi, Yu, Barrett‐Lennard, & Powles, 2008). Short‐ and long‐distance gene flow may also be mediated by seed movement on agricultural machinery and vehicles, or over short distances by wind or animals. The higher levels of admixture detected in this study compared to studies of other herbicide‐resistant weeds (Kuester et al., 2015; Okada et al., 2013, 2014) likely relate to the outcrossing nature of *L. perenne*. Spread of resistance alleles through gene flow could also result in populations and individuals containing multiple mechanisms of glyphosate resistance.

Successful management of glyphosate‐resistant *L. perenne* populations in perennial cropping systems will likely require implementation of integrated pest management programs that include chemical and nonchemical techniques to not only control currently resistant plants, but reduce the intensity of selection pressure for future independent origins of resistance and limit the spread of resistance through gene flow. Increasing tillage where possible, mowing to reduce seed set, applying herbicide alternatives to glyphosate, or applying glyphosate in mixtures with other herbicides may help prevent or delay future origins of resistance by reducing population sizes and reducing the selection pressure of glyphosate in these systems. To limit spread of resistance through gene flow, cleaning weed seed from equipment and shoes moved between infested fields may help reduce long‐distance seed transfer. Mowing or tillage may also reduce short‐distance gene flow through pollen dispersal by reducing the number of flowers resistant plants produce, although these management techniques may not be able to entirely eliminate pollen production. It is not yet known whether a fitness cost is associated with glyphosate resistance in these *L. perenne* populations, and whether the frequency of resistance would be maintained in the absence of continued glyphosate use, as no fitness studies have yet been conducted in California populations of *L. perenne* and fitness costs associated with herbicide resistance vary depending on mechanism, the measure of fitness used, and genetic background of the population (Giacomini, Westra, & Ward, 2014; Preston et al., 2009; Vila‐Aiub, Neve, & Powles, 2009). If any of the mechanisms of resistance present in the sampled area do confer a fitness cost, discontinuing use of glyphosate could result in a gradual decrease in the frequency of resistant individuals in populations. However, glyphosate is still effective on many other weed species, and will likely continue to be used in weed management in perennial crops. If gene flow between populations acts to produce populations containing multiple separate mechanisms of resistance, this could complicate their management if two mechanisms act additively resulting in plants with a very high level of resistance. Separate mechanisms of resistance may also respond differently to management if they confer different fitness penalties in the absence of continued selective pressure by glyphosate.

The results of this study show that glyphosate resistance is common in populations of *L. perenne* in orchards and vineyards of northwest California. In addition, microsatellite marker analyses revealed very high genetic diversity. All populations contained high diversity regardless of the frequency of resistant plants, contrary to what might be expected in populations undergoing strong selection for resistance. Genetic variation among populations and counties was low, in accordance with expectations for widespread and common species with highly outcrossing mating systems. The low genetic differentiation among populations and counties makes it difficult to draw conclusions about the specific origins and routes of spread of glyphosate resistance in northwest California from microsatellite variation. However, we can conclude that there were multiple origins of resistance in the region. In addition, there is potential for future spread of the resistance trait through gene flow, based on the high genetic diversity observed within all populations, the relatively low genetic differentiation among populations and counties, and the considerable admixture among populations detected in this study.

## Data Archiving Statement

Microsatellite genotype data have been archived in the Dryad Digital Repository: https://doi.org/10.5061/dryad.gs27k.
