# Supplementary material for: Genetic diversity and structure of Lolium perenne ssp. multiflorum in California vineyards and orchards indicate potential for spread of herbicide resistance via gene flow
Source: Evol Appl. 2017 Apr 18;10(6):616–29. doi: 10.1111/eva.12478 (PMC5469165; doi:10.1111/eva.12478)
Supplement: Supplementary file 1 [file EVA-10-616-s001.docx]

**Table S1:** Standard and adjusted F_ST_ values using Weir’s global estimate with and without ENA correction for null alleles.

| Locus | F_ST_ not using ENA | F_ST_ using ENA |
| --- | --- | --- |
| b1b1 | 0.0476 | 0.0379 |
| b1b3 | 0.0332 | 0.0313 |
| b3b1 | 0.0151 | 0.0117 |
| b3b8 | 0.0202 | 0.0181 |
| b3c5 | 0.0877 | 0.0762 |
| b1a8 | 0.0420 | 0.0386 |
| b4d3 | 0.0512 | 0.0491 |
| b5d12 | 0.0150 | 0.0145 |
| pr3 | 0.0133 | 0.0165 |
| 14C9-1 | 0.0061 | 0.0079 |
| 14C9-2 | 0.0112 | 0.0081 |
| 44A7 | 0.0151 | 0.0108 |
| All loci | 0.0289 | 0.0264 |
