# Supplementary material for: Genetic diversity and structure of Lolium perenne ssp. multiflorum in California vineyards and orchards indicate potential for spread of herbicide resistance via gene flow
Source: Evol Appl. 2017 Apr 18;10(6):616–29. doi: 10.1111/eva.12478 (PMC5469165; doi:10.1111/eva.12478)
Supplement: Supplementary file 2 [file EVA-10-616-s002.pdf]

Table S2. P-value results of log likelihood ratio tests for linkage disequilibrium between each pair of loci in each population.

		Population													
Locus 1	Locus 2	1	2	3	4	6	7	8	9	10	11	12	13	14	15
b1b1	b1b3	1.000	0.838	0.969	1.000	0.118	0.409	0.019	0.450	0.769	0.685	1.000	0.236	0.655	0.833
b1b1	b3b1	1.000	0.028	0.864	0.084	0.846	0.160	1.000	0.300	0.237	0.401	1.000	0.097	0.108	0.872
b1b3	b3b1	1.000	0.552	0.410	0.778	1.000	1.000	0.298	0.567	1.000	0.921	1.000	0.859	0.375	0.188
b1b1	b3b8	1.000	0.487	0.114	0.087	1.000	1.000	0.268	0.624	0.509	0.834	1.000	0*	0.718	0.056
b1b3	b3b8	1.000	0.203	1.000	1.000	1.000	0.777	0.019	0.231	0.837	0.473	1.000	0.237	0.491	0.096
b3b1	b3b8	1.000	0.238	1.000	0.244	0.375	1.000	1.000	0.544	1.000	0.288	0.068	0.242	1.000	1.000
b1b1	b3c5	0.876	0.290	0.434	1.000	0.128	0.164	0.003	0.539	0.895	0.115	0.286	0.908	0.925	0.757
b1b3	b3c5	0.337	0.265	0.414	1.000	0.268	0.307	0.588	0.760	0.148	0.258	1.000	1.000	0.543	0.149
b3b1	b3c5	0.652	0.696	0.093	1.000	0.504	1.000	0.814	0.077	1.000	0.377	0.423	0.201	0.967	0.330
b3b8	b3c5	0.675	0.749	0.896	1.000	1.000	1.000	0.507	0.914	0.263	0.630	0.340	0.026	0.583	1.000
b1b1	b4d3	1.000	0.332	0.024	1.000	0.860	0.577	0.359	1.000	0.454	0.408	0.259	0.034	0.268	0.692
b1b3	b4d3	0.539	0.849	0.988	0.820	0.117	0.147	0.940	0.087	0.558	0.048	0.781	0.909	0.583	0.274
b3b1	b4d3	0.049	0.314	0.878	1.000	0.423	0.117	0.111	0.762	0.088	0.618	1.000	0.327	0.882	0.786
b3b8	b4d3	1.000	0.693	0.352	1.000	0.481	0.339	1.000	1.000	0.907	0.948	1.000	0.424	0.294	1.000
b3c5	b4d3	0.210	0.978	0.786	0.249	0.577	0.086	0.592	0.111	0.731	0.037	1.000	0.037	0.655	1.000
b1b1	b5d12	0.836	0.594	0.576	1.000	0.925	0.564	0.252	0.397	0.102	0.239	1.000	0.053	0.819	0.145
b1b3	b5d12	0.728	0.269	0.591	0.052	0.870	0.054	0.580	0.338	0.528	0.750	0.536	0.725	0.676	0.942
b3b1	b5d12	1.000	0.662	0.519	1.000	0.003	0.052	0.821	0.445	1.000	0.896	0.537	0.003	0.725	0.404
b3b8	b5d12	0.712	0.496	0.644	1.000	1.000	0.702	0.005	0.862	0.601	0.379	0.512	0.094	0.317	0.038
b3c5	b5d12	0.559	0.421	0.270	0.476	0.259	0.457	0.240	0.109	0.770	0.780	0.028	0.018	0.319	0.539
b4d3	b5d12	0.366	0.363	0.483	0.856	0.980	0.382	0.954	0.908	0.526	0.077	0.944	0.035	0.651	0.725
b1b1	b1a8	1.000	0.581	0.802	1.000	1.000	0.994	0.590	0.742	1.000	0.115	1.000	0.040	0.066	0.396
b1b3	b1a8	0.144	0.590	1.000	1.000	1.000	0.760	0.352	0.564	0.830	0.203	1.000	0.462	0.432	0.954
b3b1	b1a8	1.000	1.000	1.000	1.000	1.000	0.655	0.331	0.692	1.000	0.499	1.000	0.048	1.000	0.019
b3b8	b1a8	0.023	0.210	1.000	1.000	1.000	0.442	0.189	1.000	0.032	0.506	1.000	0.127	0.036	1.000
b3c5	b1a8	0.513	0.390	0.396	0.430	0.454	0.722	1.000	0.984	0.790	0.680	0.336	0.599	0.502	0.361
b4d3	b1a8	0.700	1.000	0.602	0.401	0.903	0.560	0.427	0.198	0.655	0.345	1.000	1.000	0.029	1.000
b5d12	b1a8	0.705	0.445	0.847	0.450	0*	0.768	1.000	0.950	0.277	0.206	0.038	0.137	0.375	0.986
b1b1	pr3	0.751	0.916	0.053	1.000	0.859	0.970	0.836	1.000	0.616	0.129	1.000	0.532	0.058	0.335
b1b3	pr3	0.443	0.847	0.824	0.930	0.445	0.677	0.570	0.180	0.575	0.845	0.297	0.119	0.009	0.247
b3b1	pr3	1.000	1.000	0.290	1.000	0.589	1.000	0.834	1.000	1.000	0.633	1.000	0.232	0.157	0.760
b3b8	pr3	1.000	1.000	0.006	0.298	0.629	0.517	0.615	0.604	0.810	0.752	1.000	0.714	0.937	1.000
b3c5	pr3	0.216	0.519	0.449	0.718	0.103	0.158	0.309	0.967	0.055	0.519	1.000	0.310	0.848	0.323
b4d3	pr3	0.693	0.620	0.876	0.808	0.080	0.758	0.648	0.701	0.427	0.929	0.434	0.864	0.946	0.776

*: Significant following Bonferroni correction.

Table S2. P-value results of log likelihood ratio tests for linkage disequilibrium between each pair of loci in each population.

		Population													
Locus 1	Locus 2	1	2	3	4	6	7	8	9	10	11	12	13	14	15
b5d12	pr3	0.505	0.867	0.738	0.081	0.911	0.019	0.629	0.435	0.284	0.460	1.000	0.036	0.404	0.911
b1a8	pr3	1.000	1.000	0.046	0.187	0.964	0.951	0.005	0.408	1.000	0.130	1.000	0.617	0.429	0.868
b1b1	14c9-1	1.000	0.458	1.000	1.000	0.378	0.918	0.417	0.446	0.047	0.634	1.000	0.022	0.081	0.542
b1b3	14c9-1	1.000	0.841	0.775	1.000	0.217	0.588	0.745	0.809	0.927	0.314	0.726	0.361	0.074	0.345
b3b1	14c9-1	1.000	1.000	1.000	1.000	1.000	1.000	1.000	0.601	1.000	1.000	1.000	0.034	0.784	0.833
b3b8	14c9-1	1.000	1.000	1.000	1.000	1.000	1.000	1.000	1.000	0.656	0.807	1.000	0.043	0.284	0.092
b3c5	14c9-1	0.306	0.067	0.777	1.000	0.796	0.114	1.000	0.026	0.776	0.740	1.000	0*	0.103	1.000
b4d3	14c9-1	0.430	1.000	1.000	1.000	0.006	0.022	0.377	0.649	0.817	0.439	1.000	0.449	0.578	1.000
b5d12	14c9-1	1.000	0.899	1.000	1.000	1.000	0.959	0.903	0*	0.985	0.111	1.000	0.006	0.344	0.372
b1a8	14c9-1	1.000	1.000	0.306	1.000	0.451	0.794	1.000	0.347	1.000	1.000	1.000	0.021	0.215	1.000
pr3	14c9-1	0.181	1.000	1.000	1.000	0.531	0.061	1.000	1.000	1.000	1.000	1.000	0.016	0.612	0.832
b1b1	14c9-2	0.882	0.200	0.105	1.000	0.830	0.340	0.002	0.128	0.979	0.519	0.433	0.282	0.747	0.168
b1b3	14c9-2	0.291	0.248	0.906	0.953	0.935	0.354	0.802	0.132	0.010	0.611	0.972	0.115	0.890	0.079
b3b1	14c9-2	0.782	1.000	1.000	0.305	0.042	0.241	0.791	0.135	1.000	0.745	1.000	0.404	0.709	1.000
b3b8	14c9-2	1.000	0.661	1.000	1.000	0.073	0.678	0.878	1.000	0.756	0.820	1.000	0.229	1.000	0.551
b3c5	14c9-2	0.256	0.925	0.734	0.647	0.657	0.936	0.754	0.204	0.097	0.075	0.779	0.832	0.256	1.000
b4d3	14c9-2	0.208	0.266	0.252	1.000	0.038	0.818	0.332	0.691	0.301	0.746	0.597	0.983	0.652	0.766
b5d12	14c9-2	0.231	0.969	0.602	0.819	0.287	0.108	0.115	0.239	0.671	0.109	0.557	0.785	0.827	0.523
b1a8	14c9-2	0.945	1.000	1.000	1.000	0.870	0.915	0.771	0.973	0.669	0.997	0.564	0.315	1.000	1.000
pr3	14c9-2	0.232	0*	0.439	1.000	0.403	0.955	0.333	0.989	0.335	0.839	1.000	0.932	0.650	0.523
14c9-1	14c9-2	0.707	1.000	0.536	0.176	0.668	0.277	0.427	0*	0.965	0.463	0.676	0.008	0.073	0.516
b1b1	44a7	1.000	1.000	0.268	1.000	0.504	1.000	0.598	1.000	0.695	0.707	1.000	0.195	0.060	0.253
b1b3	44a7	0.035	0.711	0.235	1.000	0.579	1.000	0.571	0.163	0.126	0.620	0.144	0.565	0.146	0.132
b3b1	44a7	1.000	1.000	0.432	1.000	0.135	1.000	1.000	1.000	1.000	0.091	1.000	0.177	0.157	0.109
b3b8	44a7	1.000	1.000	1.000	1.000	1.000	1.000	1.000	0.364	1.000	1.000	1.000	0.605	0.309	0.222
b3c5	44a7	0.115	1.000	0.120	0.192	0.726	1.000	0.826	0.671	0.828	0.697	1.000	0.467	1.000	0.738
b4d3	44a7	1.000	1.000	0.616	1.000	0.801	1.000	0.204	1.000	0.080	0.900	1.000	1.000	0.573	1.000
b5d12	44a7	0.294	0.723	0.394	0.190	0.861	0.004	0.164	0.827	0.654	0.857	1.000	0.116	0.459	0.586
b1a8	44a7	1.000	1.000	1.000	0.014	0.281	0.649	1.000	1.000	0.483	0.669	1.000	0.160	0.060	0*
pr3	44a7	1.000	1.000	0.776	1.000	0.345	1.000	0.675	0.129	0.916	0.779	0.246	0.415	0.023	0.455
14c9-1	44a7	1.000	1.000	0.647	1.000	1.000	0.248	0.317	1.000	1.000	0.355	1.000	0.072	0*	0.711
14c9-2	44a7	1.000	1.000	0.648	1.000	0.914	0.035	0.949	1.000	0.291	0.150	1.000	0.170	0.887	0.890

*: Significant following Bonferroni correction.
