# Supplementary material for: Genetic diversity and structure of Lolium perenne ssp. multiflorum in California vineyards and orchards indicate potential for spread of herbicide resistance via gene flow
Source: Evol Appl. 2017 Apr 18;10(6):616–29. doi: 10.1111/eva.12478 (PMC5469165; doi:10.1111/eva.12478)
Supplement: Supplementary file 3 [file EVA-10-616-s003.pdf]

Table S3. Calculated pairwise FST values for population comparisons.

	1	2	3	4	6	7	8	9
1		NS	**	NS	NS	*	NS	NS
2	0.012		NS	NS	NS	NS	NS	NS
3	0.018	-0.002		NS	NS	NS	NS	*
4	0.012	0.009	0.012		NS	NS	NS	NS
6	0.016	0.021	0.026	0.004		NS	NS	NS
7	0.028	0.020	0.015	0.004	0.001		NS	NS
8	0.028	0.018	0.027	0.012	0.013	0.012		NS
9	0.032	0.029	0.045	0.015	0.013	0.017	0.017	
10	0.043	0.027	0.034	0.029	0.011	0.018	0.025	0.029
11	0.041	0.030	0.037	0.024	0.009	0.005	0.015	0.018
12	0.017	0.024	0.018	0.019	0.004	0.007	0.013	0.020
13	0.035	0.042	0.045	0.033	0.034	0.039	0.034	0.035
14	0.027	0.056	0.058	0.051	0.035	0.053	0.058	0.047
15	0.037	0.039	0.045	0.028	0.028	0.039	0.039	0.043

Values in lower left triangle are FST values. Upper right triangle indicates significance of those comparis

10	11	12	13	14	15
**	**	NS	**	**	**
NS	*	NS	**	**	*
NS	*	NS	**	**	**
**	**	NS	**	**	**
NS	NS	NS	NS	**	**
NS	NS	NS	**	**	**
*	NS	NS	**	**	**
**	NS	NS	**	**	**
	**	NS	**	**	**
0.017		NS	**	**	**
0.019	0.018		NS	**	NS
0.037	0.056	0.005		*	NS
0.057	0.063	0.025	0.030		**
0.046	0.058	0.008	0.012	0.027	

sons. NS: not significant. *: $P < 0.05$. **: $P < 0.01$.
