# Supplementary material for: Genetic diversity and structure of Lolium perenne ssp. multiflorum in California vineyards and orchards indicate potential for spread of herbicide resistance via gene flow
Source: Evol Appl. 2017 Apr 18;10(6):616–29. doi: 10.1111/eva.12478 (PMC5469165; doi:10.1111/eva.12478)
Supplement: Supplementary file 4 [file EVA-10-616-s004.docx]

**Supplemental Information**

**Microsatellite genotyping, null alleles and data independence**

The presence of null alleles, alleles that are present at a microsatellite locus but not detected during genotyping usually due to sequence variation, can cause individuals heterozygous for a null allele to appear homozygous for the detected allele, and bias estimates of genetic diversity and genetic differentiation. Based on comparisons of observed heterozygosity for each allele at a locus with the expected number of heterozygotes for those alleles, null alleles were detected at 8 of the 12 loci by the software Microchecker version 2.2.3 (Van Oosterhout et al. 2004). To determine the impact of these null alleles on estimates of genetic differentiation, standard estimates of F_ST_ (Weir 1996) were compared with an adjusted F_ST_ using the ‘excluding null alleles’ (ENA) method of removing null alleles and calculating F_ST_ based only on detected alleles, thus removing the bias introduced by the presence of null alleles, using FreeNa software (Chapuis and Estoup 2007) with 1000 bootstrap replicates. The standard F_ST_ value of 0.0289 (95% CI: 0.0173-0.0428) and adjusted F_ST_ using the ENA method of 0.0264 (95% CI: 0.0159-0.0387) were significantly different (Table S1). However, both estimates of F_ST_ give the same trend across loci (Table S1), indicating that both methods will yield similar results in further analyses of genetic differentiation and the presence of null alleles is unlikely to influence conclusions drawn from those analyses. Non-adjusted F_ST_ values are used in all further analyses.

Among 923 pairwise comparisons of loci and populations, eight pairs of loci, each in a single population, are in linkage disequilibrium with each other following Bonferroni correction (Table S2).
